# Translation, cross-cultural adaptation, and psychometric validation of the Chinese version of the Caregiver Contribution to Self-Care of Coronary Heart Disease Inventory

**DOI:** 10.3389/fpubh.2026.1751188

**Published:** 2026-03-30

**Authors:** Ziming He, Yuxuan Zhang, Tingting Zou, Yunbin Teng, Yuanyuan Zhang, Tingting Song, Tong Yue, Junping Li, Sainan Xi

**Affiliations:** 1 School of Nursing, Dalian University, Dalian, Liaoning, China; 2 Dalian No.3 People’s Hospital, Dalian, Liaoning, China; 3 Dalian University Affiliated Xinhua Hospital, Dalian, Liaoning, China

**Keywords:** caregiver contribution, confirmatory factor analysis, coronary heart disease, cross-cultural adaptation, instrument validation

## Abstract

**Objective:**

This study aimed to translate the Caregiver Contribution to Self-Care of Coronary Heart Disease Inventory, version 3a (CC-SC-CHDI v3a), into Chinese and evaluate its psychometric properties after cultural adaptation.

**Methods:**

Between December 2024 and April 2025, a cross-sectional survey was conducted with 278 primary caregivers of patients with coronary heart disease, recruited from cardiology departments in two hospitals in Dalian, China. Translation was based on the Brislin model, which involved forward–backward translation, expert panel assessment, and a pilot test with 30 caregivers. Psychometric evaluation included item analysis, assessment of content validity, convergent validity, concurrent validity, and confirmatory factor analysis (CFA). Reliability was assessed through internal consistency (Cronbach’s *α*).

**Results:**

The Chinese version of the CC-SC-CHDI v3a demonstrated high content validity, with item-level CVI values ranging from 0.90 to 1.00 and scale-level CVI values between 0.97 and 1.00. All items had critical ratios exceeding 3.0 and item–total correlations above 0.4. CFA results indicated a satisfactory model fit (*χ*^2^/df < 3; GFI, CFI, NFI, IFI > 0.90; RMSEA < 0.08). Cronbach’s *α* values for the three subscales were 0.821, 0.870, and 0.840.

**Conclusion:**

The culturally adapted Chinese version of the CC-SC-CHDI v3a is a reliable and valid tool for assessing caregiver contributions to self-care in Chinese patients with coronary heart disease.

## 1 Introduction

Coronary heart disease (CHD) has become a major global public health concern, imposing a substantial burden on healthcare systems and significantly affecting patients’ quality of life. It is estimated that approximately 7 million deaths worldwide each year are attributable to CHD (1). In China, driven by population aging and lifestyle changes, both the prevalence and mortality of CHD continue to rise. According to the China Cardiovascular Health and Disease Report 2023, the number of CHD cases in China has increased by about 30% over the past decade, making it one of the most prevalent chronic diseases threatening population health (2).

Current therapeutic strategies for CHD mainly focus on symptom relief, improvement of myocardial ischemia, and reduction of short-term adverse cardiovascular events. However, these treatments cannot fundamentally reverse the progression of coronary atherosclerosis. Consequently, patients remain at long-term risk of adverse outcomes, including unstable angina, myocardial infarction (MI), heart failure, and thrombotic events (3). In this context, effective long-term management has become a key challenge in CHD care. Self-care has been recognized as an essential component of chronic disease management, contributing to reduced recurrence, delayed disease progression, and improved quality of life (4). Self-care refers to the process through which individuals maintain health by engaging in health-promoting behaviors and disease management activities (5). Evidence suggests that effective self-care practices can reduce complications, enhance patients’ participation in health management, and decrease healthcare resource utilization (6).

According to the Theory of Self-Care in Chronic Illness, self-care is a dynamic process consisting of three interrelated components: self-care maintenance, self-care monitoring, and self-care management. Support from family members and social networks plays an important role in facilitating this process (7). Caregivers—most commonly spouses, adult children, or other family members—often assume substantial responsibilities in assisting patients with disease management. In addition to supporting daily activities, caregivers provide emotional encouragement, health-related guidance, and assistance during sudden health events (8–10).

The Theory of Caregiver Contributions further introduces the concept of caregiver contributions to self-care, emphasizing that caregivers’ time investment, behavioral assistance, and social support can significantly influence patients’ self-care behaviors in chronic disease management (11). In recent years, increasing attention has been paid to the involvement of caregivers in patient self-care. Researchers have advocated incorporating caregivers into discharge education and self-care intervention programs to improve long-term disease management outcomes (12).

Empirical studies have demonstrated that when patients participate in interventions such as medication adherence management, exercise training, symptom monitoring, and symptom management together with informal caregivers, their self-care levels can be significantly improved (13). Moreover, caregiver participation in lifestyle intervention programs has been shown to enhance patients’ self-efficacy, promote healthier behaviors, and reduce caregiver burden (14, 15). Despite these advances, most existing studies focus primarily on patient health outcomes or caregivers’ psychosocial status, while relatively little attention has been given to systematically measuring caregivers’ specific contributions to patient self-care (16, 17).

Accurate assessment of caregiver contributions is therefore important for understanding the role of family support in chronic disease management. Reliable measurement tools are needed to quantify the extent to which caregivers support patients’ self-care behaviors and to facilitate the evaluation of family-centered interventions (18). Bolgeo et al. (19) developed the Caregiver Contribution to Self-Care of Coronary Heart Disease Inventory (CC-SC-CHDI v3a) and demonstrated its satisfactory reliability and validity among caregivers of patients with CHD (18). However, culturally adapted and psychometrically validated instruments suitable for the Chinese context are currently lacking, which limits the systematic evaluation of family caregivers’ roles in CHD self-management in China.

Therefore, this study aimed to translate and culturally adapt the English version of the CC-SC-CHDI v3a into Chinese and to examine its reliability and validity. To our knowledge, this is the first study to conduct the translation and psychometric validation of this instrument among mainland Chinese populations. The validated Chinese version of the scale may provide a reliable tool for assessing caregivers’ contributions to self-care in patients with CHD and offer a measurement basis for future family-centered chronic disease management interventions.

## 2 Methods

### 2.1 Design and participants

This study aimed to evaluate the Caregiver Contribution to Self-Care among caregivers of patients with CHD, conducted between December 2024 and April 2025 in the cardiology departments of two hospitals in Dalian, China, using a cross-sectional study design. The primary survey was administered by researchers in outpatient clinics and wards using face-to-face paper questionnaires with caregivers who met the study’s inclusion criteria. The sample size was determined based on the number of items in the CC-SC-CHDI v3a, which includes 24 items. A sample size 10 times the number of items was deemed appropriate, resulting in an initial target of 240 participants. To account for a potential 20% dropout rate, the final sample size was increased to 288.

Caregiver inclusion criteria were as follows: (1) Chinese adults aged 18 years or older; (2) Primary caregivers of patients with CHD who spend 10 or more hours per week accompanying, caring for, or supervising the patient; (3) Willingness to participate and cooperate in the study; (4) Ability to complete the questionnaire in Mandarin. Exclusion criteria were as follows: (1) Individuals unable to communicate verbally or in writing; (2) Those with severe cognitive impairments; (3) Individuals with severe psychological or psychiatric disorders.

### 2.2 Instruments

#### 2.2.1 General information

The general demographic questionnaire for caregivers in this study was custom-designed. It collected the following demographic information: age, sex, educational level, health status, residence, living arrangement with the CHD patient, relationship to the CHD patient, patient’s dependency on the caregiver, and years of caregiving.

#### 2.2.2 The caregiver contribution to self-Care of Coronary Heart Disease Inventory (CC-SC-CHDI v3a)

The CC-SC-CHDI v3a was developed by Bolgeo et al. (19), adapted from the Self-Care of Coronary Heart Disease Inventory by Dickson et al. (20), to assess the involvement of caregivers (e.g., family members or other informal caregivers) in the self-care behaviors of patients with CHD. It consists of three scales, comprising a total of 24 items. The self-care maintenance scale (9 items) includes two dimensions: illness-related behaviors and health-promoting behaviors. Responses are rated on a Likert scale from 1 to 5, with “never or rarely” scored as 1 and “always or daily” scored as 5. The self-care monitoring scale (7 items) consists of a single dimension focused on monitoring behaviors. Responses are rated on a Likert scale from 1 to 5, using the same scoring system as described above. Two separate items on symptom recognition are rated on a Likert scale from 0 to 5, with “I did not recognize the symptom” scored as 0, “not quickly” as 1, and “very quickly” as 5. The self-care management scale (6 items) includes two dimensions: consulting behaviors and problem-solving behaviors. Five items are rated on a Likert scale from 1 to 5, with “not likely” scored as 1 and “very likely” scored as 5. The final item is rated on a Likert scale from 0 to 5, with “I did not do anything” scored as 0, “not sure” as 1, and “very sure” as 5. Scores for all three scales are converted into standardized scores ranging from 0 to 100, with higher scores indicating greater caregiver contribution. The internal consistency indices for the scales ranged from 0.73 to 0.90 (19).

#### 2.2.3 The caregiver self-efficacy in contributing to patient self-care (CSE-CSC) scale

The CSE-CSC scale was developed by De Maria et al. (21), and the Chinese version was translated by Lv et al. (22). This scale is designed to assess caregivers’ self-efficacy in patient self-care, with 10 items rated on a scale from 1 = “not confident” to 5 = “very confident.” The total score is standardized on a scale from 0 to 100, with higher scores indicating greater caregiver self-efficacy in contributing to patient self-care. The reliability of the scale includes a Cronbach’s *α* coefficient of 0.87, test–retest reliability of 0.83, and split-half reliability of 0.90.

#### 2.2.4 Family caregiver task inventory (FCTI)

The FCTI was originally developed by Clark and Rakowski (23). The Chinese version was subsequently translated by scholars Lee and Mok (24). This scale consists of 25 items and is divided into five dimensions: Learning to cope with new roles (5 items), Providing care based on the care receiver’s needs (5 items), Managing one’s emotional needs (5 items), Appraising supportive resources (5 items), and Balancing caregiving needs with one’s own needs (5 items). The scale uses a 3-point Likert scale, with scores of 0, 1, and 2 representing “not difficult,” “difficult,” and “extremely difficult,” respectively. The total score ranges from 0 to 50, with higher scores indicating a greater level of caregiving difficulty experienced by the caregiver. The scale demonstrates high internal consistency, with a Cronbach’s *α* coefficient of 0.93, and confirmatory factor analysis supports its reliability and validity (24).

### 2.3 Procedure

#### 2.3.1 Questionnaire translation procedure

The cross-cultural adaptation process of the inventory followed the Brislin (25) model and adhered to the guidelines established by the original authors (26). The scale translation, cross-cultural adaptation and psychometric evaluation were based on the COSMIN and other internationally recognized standards (27). The initial Chinese translation was performed by the author and an associate professor proficient in specialized English. The first draft was created through consensus after comparing and discussing the translations. This initial translation was then back-translated into English by two English-speaking professionals who were unfamiliar with the source inventory. After comparing the back-translation with the original version and making necessary revisions to reach consensus, the back-translated version was sent to the original authors for approval. Seven researchers in the field of nursing, all of whom had studied abroad, subsequently evaluated the equivalence of the source inventory and the first draft of the translation. Revisions were made based on their evaluations and feedback. After completing the above steps, the translated manuscript was assessed for content validity by 10 experts with diverse backgrounds in medicine, clinical nursing, nursing education, and psychology, and revisions were made based on their evaluations and comments. A pre-survey was subsequently conducted with 30 caregivers of patients with CHD at a tertiary hospital in Dalian, leading to the final Chinese version for the official survey.

#### 2.3.2 Data collection procedure

The first round of distribution was conducted exclusively in paper form by researchers in person within the wards and outpatient departments of two hospitals. This study used a convenience sampling method to select caregivers of patients with coronary heart disease from Dalian No.3 People’s Hospital and Dalian University Affiliated Xinhua Hospital to conduct a cross-sectional survey. The survey at Dalian No.3 People’s Hospital was conducted from February 2025 to April 2025, while the survey at Dalian University Affiliated Xinhua Hospital was conducted from February 2025 to March 2025. A total of 322 eligible participants were identified, 283 questionnaires were returned, and 278 valid questionnaires were obtained, yielding an actual participation rate of 87.9%. Informed consent was obtained from the participants by the researchers prior to distribution, and the questionnaires were collected immediately afterward.

### 2.4 Data analysis

Data analysis was conducted using SPSS 23.0 and AMOS software. Descriptive statistics, critical ratio (CR), correlation coefficient methods, confirmatory factor analysis (CFA), Cronbach’s *α* coefficient were employed for data analysis. A *p*-value of less than 0.05 was regarded as statistically significant.

#### 2.4.1 Items analysis

Each of the three scales was divided into high-score and low-score groups based on the total score, and the relationship between the two groups was analyzed to assess the discriminative ability of each scale. The critical ratio (CR) for the corrected item-total correlation were 0.3 (28). The correlation coefficient between each item and the total score was calculated for each scale. The items with a correlation coefficient (*r*) should exceed 0.4 as the reference standard (29). Items should be deleted if the Cronbach’s *α* coefficient for any scale increases after removing a specific item (30).

#### 2.4.2 Validity analysis

Content validity was primarily evaluated through expert evaluation and the calculation of the Content Validity Index (CVI) (31). It is widely accepted that an I-CVI of 0.78 or higher demonstrates strong content validity for individual items when the panel includes six or more experts, while an S-CVI greater than 0.8 indicates that the scale possesses good content validity overall (32, 33).

Convergent validity was established by examining the correlations between the CC-SC-CHDI v3a and the Chinese version of the CSE-CSC. Concurrent validity was assessed by evaluating the correlations between the CC-SC-CHDI v3a and the Chinese version of the FCTI.

Confirmatory Factor Analysis (CFA) was conducted to assess construct validity. The sample size was initially estimated based on the rule of thumb of at least 10 participants per item. In addition, methodological studies suggest that a sample size of 200 or more is generally adequate for confirmatory factor analysis and structural equation modeling (34). Therefore, the final sample size of 278 participants in this study was considered sufficient to test the proposed confirmatory factor structure model. Various model fit indices, including a chi-square/degree of freedom ratio (*χ*^2^/df) of less than 3, a Goodness of Fit Index (GFI) greater than 0.9, a Root Mean Square Error of Approximation (RMSEA) of less than 0.08, a Comparative Fit Index (CFI) greater than 0.9, a Normed Fit Index (NFI) greater than 0.9, and an Incremental Fit Index (IFI) greater than 0.9, were employed to evaluate the model’s goodness of fit (35).

#### 2.4.3 Reliability analysis

Cronbach’s *α* is used to assess internal consistency reliability. It is generally accepted that a Cronbach’s α greater than 0.70 indicates good internal consistency reliability for the scale (36).

### 2.5 Ethical approval

The study underwent ethical review at the Dalian No.3 People’s Hospital, with approval number 2024-162-001, and at Dalian University Affiliated Xinhua Hospital, under ethical review opinion number 2025-004-01. During participation, an informed consent form and written information will be provided to the participants.

## 3 Results

### 3.1 Descriptive statistics

A total of 278 valid questionnaires were collected in this study. Among the recruited caregivers (from both inpatient and outpatient departments), 156 were female (56.5%), and 120 were male (43.5%). Further details are provided in Table 1.

**Table 1:** General demography data (*n* = 278).

Factors	Group	*N*	%
Age	<40	47	17.0
	40–50	57	20.7
	50–60	75	27.2
	60–70	59	21.4
	≥70	38	13.7
Sex	Male	120	43.5
	Female	156	56.5
Education level	Primary school or below	16	5.8
	Junior high school	89	32.2
	Senior high school (or equivalent)	78	28.3
	Bachelor’s degree (or above)	93	33.7
Self-reported health status	Poor	6	2.3
	Fair	72	27.1
	Good	188	70.6
Residence	Rural	24	8.6
	Urban	254	91.4
Living arrangement with CHD patient	Different residence	98	35.3
	Same residence	180	64.7
Relationship with CHD patient	Spouse	92	33.1
	Child	148	53.2
	Other	38	13.7
Patient’s dependency on caregiver	Not dependent at all	57	21.2
	Mildly dependent	169	62.8
	Mostly dependent	29	10.8
	Completely dependent	14	5.2
Years of caregiving	<2 year	65	24.2
	2–5 years	77	28.6
	5–10 years	62	23.0
	≥10 years	65	24.2

### 3.2 Cross-cultural adaption and pre-survey results

The results of the equivalence evaluation of the Chinese version of the CC-SC-CHDI v3a in this study indicated that the percentage of positive ratings for the items, indicating “quite equivalent” or “most equivalent,” ranged from 85.7 to 100%. This demonstrated a high level of consistency in the scores of each item (37). Based on the equivalence evaluation, expert content validity assessment, and discussions within the research team, 12 revisions and improvements were implemented. Thirty caregivers were pre-surveyed for this study, and no modifications to the questionnaire were suggested.

### 3.3 Items analysis

The item analysis results for the Chinese version of the CC-SC-CHDI v3a across the three scales are shown in Table 2. The Critical Ratio (CR) values for all items were above 3, and the Item-Total Correlation (*r*) for all items was greater than 0.4.

**Table 2:** Item analysis for the Chinese version of the CC-SC-CHDI v3a (*n* = 278).

Number	Item	Critical ratio (CR)	Item-total correlation (*r*)
Self-care maintenance scale			
Illness-related behaviors			
1	Keep appointments with the healthcare provider?	9.837	0.569
2	Take aspirin or other blood thinner?	12.727	0.613
5	Take prescribed medicines without missing a dose?	13.485	0.641
Health-promoting behaviors			
3	Do something to relieve stress (e.g., medication, yoga, music)?	12.680	0.638
4	Do physical activity (e.g., take a brisk walk, use the stairs)?	12.169	0.637
6	Ask for low fat items when eating out or visiting others?	19.883	0.752
7	Try to avoid getting sick (e.g., flu shot, wash your hands)?	14.380	0.730
8	Eat fruits and vegetables?	14.185	0.684
9	Avoid cigarettes and/or smokers?	9.687	0.489
Self-care monitoring scale			
Monitoring behaviors			
10	Monitor your condition?	17.034	0.744
11	Pay attention to changes in how they feel?	16.331	0.735
12	Check the blood pressure?	16.659	0.761
13	Monitor whether they tire more than usual doing normal activities?	13.032	0.658
14	Monitor for medication side-effects?	20.628	0.805
15	Monitor for symptoms?	20.625	0.818
16	Monitor body weight?	17.031	0.742
Self-care management scale			
Consulting behaviors			
22	Call the healthcare provider for guidance	15.970	0.582
23	Tell the healthcare provider about the symptom at the next office visit	11.893	0.681
*Problem-solving behaviors*			
19	Change the activity level (slow down, rest)	16.844	0.738
20	Take an aspirin	26.535	0.830
21	Take a medicine to make the symptom decrease or go away	19.423	0.793
24	Did the treatment you used make them feel better?	14.730	0.718

### 3.4 Validity analysis

#### 3.4.1 Content validity analysis

The item-level content validity index (I-CVI) ranged from 0.9 to 1.0, exceeding the threshold of 0.78, while the scale-level content validity index (S-CVI) for the three scales ranged from 0.97 to 1.0, surpassing the 0.8 threshold. This indicates strong content validity.

#### 3.4.2 Convergent validity analysis

This study examined the Pearson correlation coefficients between self-efficacy (CSE-CSC, Chinese version) and the three subscales of the CC-SC-CHDI v3a scale (self-care maintenance, self-care monitoring, and self-care management). The analysis revealed a significant and strong positive correlation between self-efficacy and self-care maintenance (*r* = 0.400, *p* < 0.01), as well as a significant positive correlation between self-efficacy and self-care monitoring (*r* = 0.459, *p* < 0.01). Self-care monitoring was also significantly positively correlated with self-care management (*r* = 0.355, *p* < 0.01), and self-efficacy was significantly positively correlated with self-care management (*r* = 0.662, *p* < 0.01).

#### 3.4.3 Concurrent validity analysis

This study examined the Pearson correlation coefficients between the total score of the Chinese version of the FCTI and the total scores of the three subscales of the CC-SC-CHDI v3a. The analysis indicated that caregivers’ overall care competence score was significantly negatively correlated with self-care maintenance (*r* = −0.139, *p* < 0.05), while no significant correlation was observed with self-care monitoring. Care competence was also significantly negatively correlated with self-care management (*r* = −0.337, *p* < 0.01). The individual dimensions of caregiving capacity showed no significant correlation with self-care monitoring but were significantly negatively correlated with self-care management (*p* < 0.01). Within caregiving capacity, the dimensions of addressing personal emotional needs and assessing family and community resources showed no significant correlation with self-care maintenance, whereas all other scales were significantly negatively correlated (*p* < 0.05). Since the FCTI scale is reverse-scored (with higher scores indicating greater caregiving difficulties), the results should be interpreted as a negative correlation between the level of caregiving difficulties and patients’ self-care abilities. In other words, fewer caregiving difficulties are associated with better self-care maintenance and management, rather than indicating a negative correlation between caregiving ability and self-care ability.

#### 3.4.4 Confirmatory factor analysis

**Figure 1:**
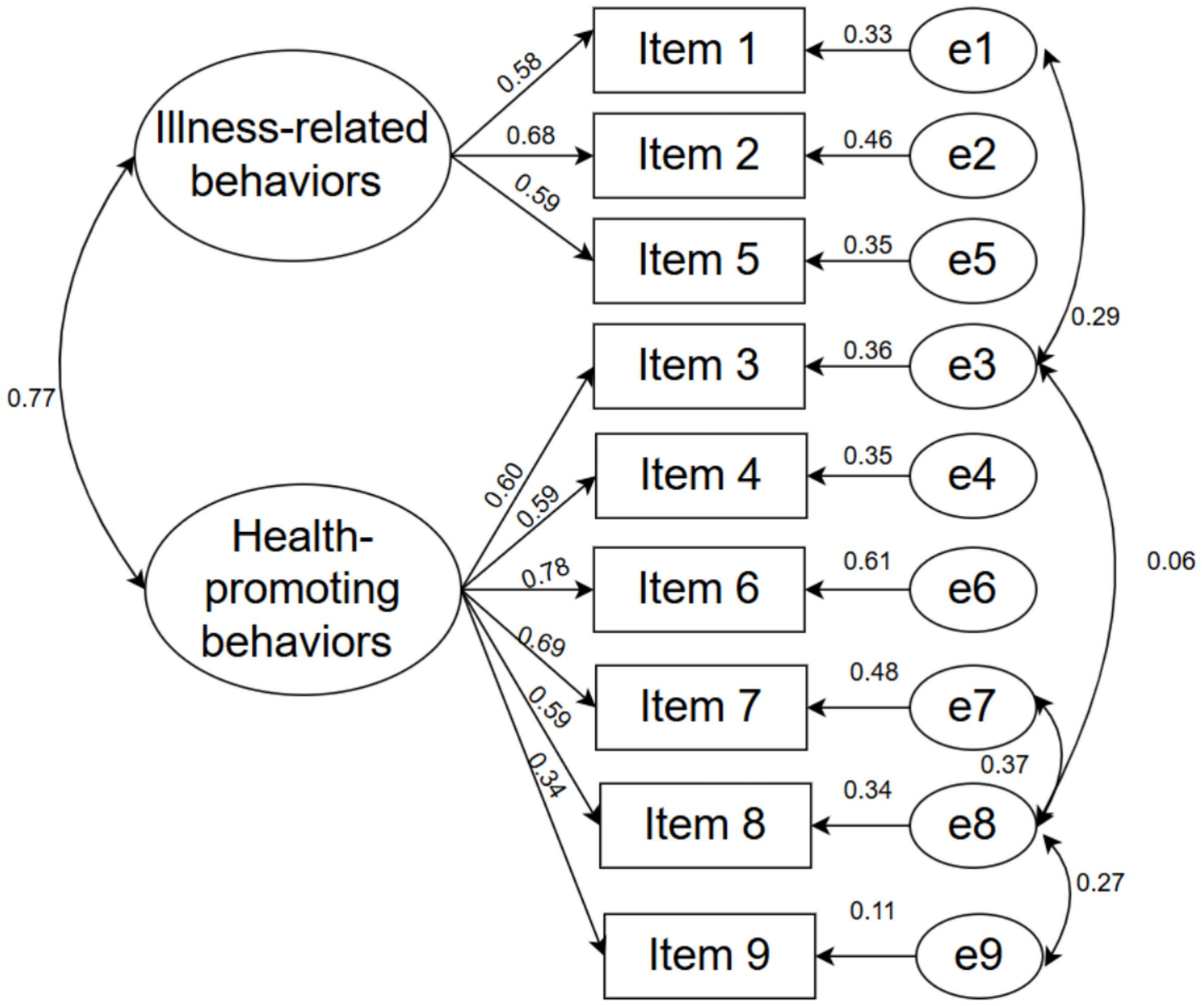
The factor structure diagram for Self-Care Maintenance Scale.

Confirmatory factor analysis (CFA) was conducted to assess the structural validity of the revised three scales. The model fit indices for the Self-Care Maintenance Scale were *χ*^2^/df = 5.832, RMSEA = 0.132, GFI = 0.907, CFI = 0.863, NFI = 0.841, and IFI = 0.865. Due to the inadequacy of these model fit indices, error correlations between items were added according to the modification indices. The model fit indices for the revised model were *χ*^2^/df = 2.608, RMSEA = 0.076, GFI = 0.957, CFI = 0.951, NFI = 0.924, and IFI = 0.952. The factor structure of the revised model for the Self-Care Maintenance Scale is illustrated in Figure 1.

**Figure 2:**
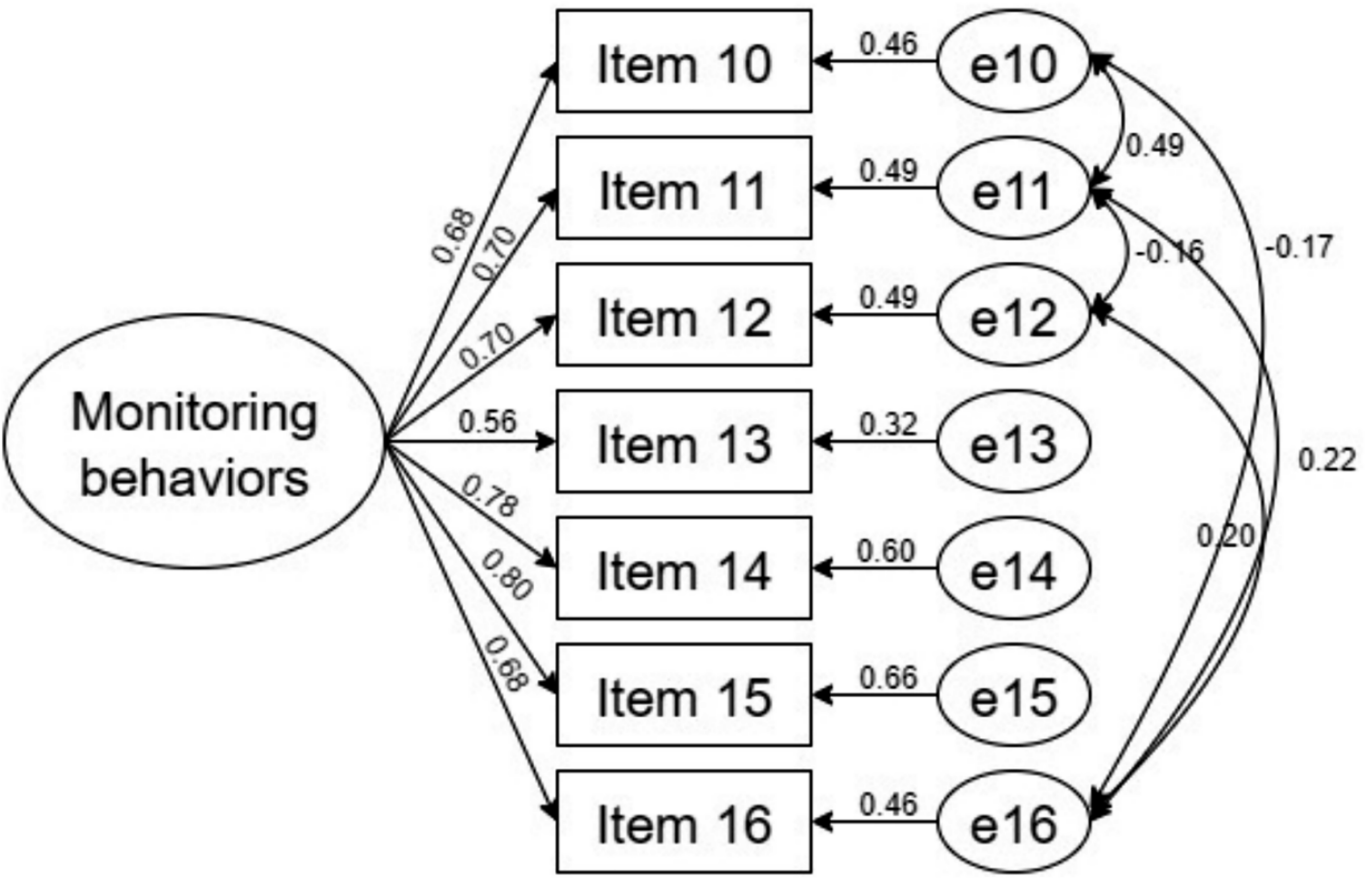
The factor structure diagram for Self-Care Monitoring Scale.

The model fit indices for the Self-Care Monitoring Scale were *χ*^2^/df = 9.120, RMSEA = 0.171, GFI = 0.874, CFI = 0.873, NFI = 0.861, and IFI = 0.874. Due to the inadequacy of these model fit indices, error correlations between items were added according to the modification indices. The model fit indices for the revised model were *χ*^2^/df = 2.265, RMSEA = 0.068, GFI = 0.979, CFI = 0.987, NFI = 0.978, and IFI = 0.987. The factor structure of the revised model for the Self-Care Management Scale is illustrated in Figure 2.

**Figure 3:**
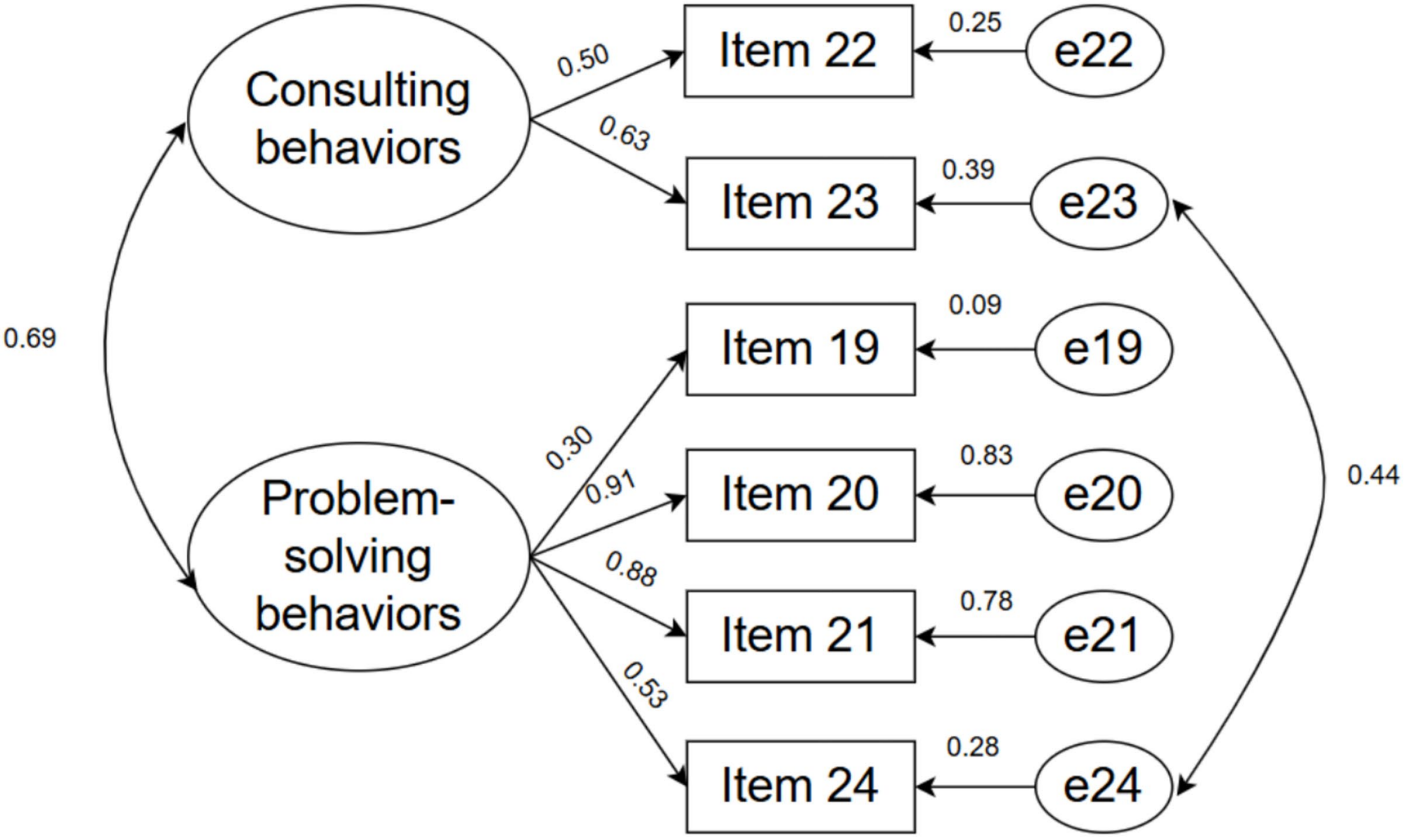
The factor structure diagram for Self-Care Management Scale.

The model fit indices for the Self-Care Management Scale were *χ*^2^/df = 18.602, RMSEA = 0.252, GFI = 0.865, CFI = 0.863, NFI = 0.858, and IFI = 0.864. Due to the inadequacy of these model fit indices, error correlations between items were added according to the modification indices. The model fit indices for the revised model were *χ*^2^/df = 2.171, RMSEA = 0.065, GFI = 0.983, CFI = 0.985, NFI = 0.972, and IFI = 0.985. The factor structure of the revised model for the Self-Care Management Scale is illustrated in Figure 3.

### 3.5 Reliability analysis

The translated versions of the Self-Care Maintenance Scale, Self-Care Monitoring Scale, and Self-Care Management Scale showed reliability coefficients of 0.821, 0.870, and 0.840, respectively.

## 4 Discussion

### 4.1 Applicability of the Chinese version of CC-SC-CHDI v3a

This study demonstrates that the Chinese version of the Caregiver Contribution to Self-Care of Coronary Heart Disease Inventory (CC-SC-CHDI v3a) possesses sound psychometric properties and is suitable for use in the Chinese context. The instrument provides a reference framework for assessing caregivers’ supportive roles in the self-care of patients with coronary heart disease (CHD). Against the backdrop of population aging and the growing burden of cardiovascular disease, caregiver involvement has become a key factor in sustaining patients’ long-term self-management and rehabilitation (37).

The Chinese version reflects certain behavioral characteristics of family caregiving within the Chinese cultural context, such as supervision of medication adherence and dietary management, which receive relatively less attention in Western contexts. Accordingly, it represents not only a successful instance of cross-cultural psychometric adaptation but also an important tool for research on family-centered cardiac care. In clinical practice, the scale can help healthcare professionals identify caregivers who require additional training or psychological support and, in turn, optimize health education and intervention programs. At the public-health level, it offers empirical support for policies and initiatives aimed at improving outcomes for both patients and families. Overall, the Chinese CC-SC-CHDI v3a contributes to advancing healthy aging and deepening family-engaged chronic disease management.

### 4.2 Item distinctiveness and cultural adaptation

The translation and cultural adaptation of the scale followed the Brislin model; forward–backward translation, expert review, and pilot testing were employed to ensure conceptual equivalence. Item-equivalence testing indicated high agreement on clarity and relevance (85.7%–100%), suggesting that the translated items are comprehensible to caregivers across diverse cultural and educational backgrounds. Item analysis showed that all items had critical ratios greater than 3.0 and item–total correlation coefficients exceeding 0.40. Item 9 (“avoid smoking and/or stay away from smokers”) has limited operability in the Chinese cultural context, as many caregivers—particularly women—lack authority to intervene in the smoking behavior of older male patients, and smoking remains socially prevalent (38). Item 22 (“call healthcare professionals for guidance”) reflects a behavior that is less common in Chinese caregiving practice, where caregivers more often seek face-to-face consultation or advice through informal social networks.

Overall, the Chinese version retains the conceptual integrity of the original while aligning more closely with Chinese family caregiving practices; the items are concise and semantically clear, facilitating use and understanding among middle-aged and older caregivers.

### 4.3 Reliability of the Chinese version

Reliability analyses indicated that all three subscales of the Chinese version of the CC-SC-CHDI v3a achieved satisfactory internal consistency, with Cronbach’s *α* coefficients of 0.821 for self-care maintenance, 0.870 for self-care monitoring, and 0.840 for self-care management, each exceeding the conventional threshold of 0.70. These results align with the α range reported in the original study by Riegel et al. (39) (0.73–0.90), indicating that the translated instrument preserves the structural coherence of the original. Notably, the self-care monitoring subscale yielded the highest α (0.870), demonstrating particularly strong internal consistency in this sample. High internal consistency suggests that the items within each subscale assess the same latent construct with minimal redundancy. Reliable measurement is essential for both research and practice, ensuring that observed differences in caregiver contribution reflect true behavioral variation rather than measurement error (40).

This pattern also reflects distinctive behavioral characteristics of Chinese caregivers in symptom observation and reporting: they tend to evaluate changes in patients’ conditions through meticulous daily observation (e.g., symptoms, diet, sleep, and psychological state). Such an “observation-centered” caregiving model is consistent with a strong sense of familial responsibility and a collectivist cultural context. Compared with Western samples, Chinese caregivers place greater emphasis on direct supervision and hands-on involvement rather than encouraging fully autonomous self-management. The high reliability of the monitoring subscale may therefore capture a core cultural feature of “close supervision and continuous care.” These findings not only support the appropriateness of the cross-cultural adaptation but also point to important directions for future intervention research.

### 4.4 Validity of the Chinese version

#### 4.4.1 Content validity

Content validity analyses indicate that the Chinese version exhibits excellent representativeness and relevance. The item-level content validity index (I-CVI) ranged from 0.90 to 1.00, and the scale-level content validity index (S-CVI) ranged from 0.97 to 1.00, both exceeding recommended standards (41). The expert panel comprised specialists in nursing, psychology, and clinical medicine, several of whom had international research experience, thereby ensuring linguistic accuracy and conceptual equivalence.

#### 4.4.2 Convergent and concurrent validity

Convergent validity analyses were consistent with theoretical expectations. Caregiver self-efficacy (CSE-CSC) showed significant positive correlations with all three subscales of the scale, with the strongest association observed for self-care management (*r* = 0.662, *p* < 0.01), indicating that higher caregiver confidence is associated with more active involvement in disease management and symptom response (42).

Concurrent validity was also supported. Caregiver competence (Family Caregiver Task Inventory) was significantly negatively correlated with self-care maintenance (*r* = −0.139, *p* < 0.05) and self-care management (*r* = −0.337, *p* < 0.01), suggesting that caregivers with heavier burden are less able to support patients’ self-care. The higher the caregiver’s score on the Self-Care Contribution Scale, the lower the perceived task difficulty on the FCTI. This result not only validates the criterion-related validity of the new scale but also empirically supports the positive correlation between caregiver contribution and caregiving capacity. This inverse relationship is consistent with prior research linking emotional exhaustion to reduced caregiving engagement (43).

#### 4.4.3 Construct validity and confirmatory factor analysis

Confirmatory factor analysis (CFA) indicated good fit for the revised model (χ^2^/df < 3, RMSEA < 0.08, and GFI, CFI, NFI, IFI all > 0.90), demonstrating a robust scale structure. The overall factor configuration was consistent with the confirmatory factor structure model, supporting the view that caregivers perform three core functions in self-care: maintenance, monitoring, and management. Item 21 (“take medication to relieve or eliminate symptoms”) showed a relatively low loading (< 0.50) on the self-care management subscale, suggesting a weaker association with the underlying construct. A plausible explanation is that caregivers generally follow physicians’ instructions closely and seldom adjust dosages independently, thereby reducing behavioral variability and its linkage to the construct. In addition, nuances of the Chinese translation may have introduced ambiguity, leading some caregivers to interpret the item as referring to their own behavior.

### 4.5 Model refinement and theoretical justification for correlated error terms

For the Self-Care Maintenance Scale, the initial model demonstrated inadequate fit. After examining the modification indices, correlations between several item error terms were added based on theoretical considerations, which significantly improved model fit (44). One possible explanation is that some items share highly similar wording and refer to closely related caregiving behaviors, such as supporting health-promoting activities and assisting with illness-related management. Items that address similar behavioral domains may contain overlapping content, which can lead to shared variance beyond the latent construct. In addition, items that appear in close proximity within the questionnaire may also introduce correlated measurement errors due to respondents’ consistent response patterns. Allowing theoretically justified correlations between measurement errors is considered acceptable in confirmatory factor analysis when items reflect closely related behavioral domains.

The Self-Care Monitoring Scale also showed unsatisfactory fit in the initial model but achieved acceptable fit after model modification guided by the modification indices (44). Monitoring behaviors generally involve observing symptoms and identifying changes in patients’ health conditions. Because these activities often occur in similar caregiving situations, certain items may share additional variance beyond the latent construct. Accounting for these theoretically plausible correlations between residuals may therefore contribute to the improved model fit.

For the Self-Care Management Scale, the initial model also demonstrated inadequate fit but reached satisfactory fit after theoretically guided model refinement. In addition, a small number of items showed relatively lower standardized factor loadings. Specifically, Item 9 in the maintenance subscale and Item 19 in the management subscale demonstrated lower loadings compared with other items. In scale validation studies, slightly lower factor loadings may still be acceptable when items remain conceptually relevant to the construct. One possible explanation is that these caregiving behaviors may be strongly influenced by physicians’ recommendations or healthcare practices, which may limit caregivers’ autonomy and reduce response variability. Subtle differences introduced during translation and cross-cultural adaptation may also influence respondents’ interpretation of certain items (45).

### 4.6 Implications for research and clinical practice

The validation of the Chinese CC-SC-CHDI v3a provides researchers and clinicians with a standardized, culturally well-adapted assessment tool. In research settings, the instrument can be used to examine associations between caregiver behaviors and patient outcomes such as adherence, symptom control, and readmission rates; notably, the high reliability of the monitoring dimension offers a robust indicator for quantifying caregiving behaviors.

In clinical practice, the scale can help healthcare professionals identify caregivers who experience difficulties with illness monitoring and medication management, thereby enabling the provision of individualized guidance and psychological support. Routine application of the instrument may also foster active family engagement in cardiac rehabilitation and disease management, ultimately improving overall health status and quality of life for both patients and caregivers.

### 4.7 Limitations and future directions

The study sample was drawn from two hospitals in Dalian, reflecting a strong regional focus that may limit the generalizability of the findings. Future research should broaden the sampling frame to include caregivers from diverse regions, cultural backgrounds, and levels of the healthcare system. Longitudinal designs are recommended to further evaluate the instrument’s temporal stability and sensitivity to intervention effects.

In addition, because Item 21 exhibited a relatively low factor loading, subsequent work should consider revising this item—while preserving the theoretical framework—or introducing medication-management indicators that are more culturally representative. The scale could also be applied to caregivers of other chronic cardiac conditions (e.g., heart failure) to assess its broader applicability.

## 5 Summary

This study demonstrates that the Chinese version of the CC-SC-CHDI v3a is a reliable and valid instrument for evaluating caregivers’ contributions to the self-care of patients with coronary heart disease. Following systematic translation and cultural adaptation, the scale retained the original confirmatory factor structure and showed excellent reliability, content validity, convergent validity, concurrent validity, and construct validity. The self-care monitoring subscale exhibited the highest reliability in the Chinese sample (*α* = 0.870), highlighting a stable pattern of daily monitoring, meticulous observation, and responsibility-driven behaviors among Chinese caregivers. This cultural feature underpins both the academic value and the practical distinctiveness of the Chinese version.

Despite a small number of items with lower factor loadings, the overall model achieved outstanding fit (χ^2^/df < 3, RMSEA < 0.08, and GFI, CFI, NFI, IFI > 0.90), indicating structural robustness and preserved theoretical integrity. The Chinese CC-SC-CHDI v3a can be widely applied in research and clinical practice to assess and enhance family engagement in CHD management, advance family-based cardiovascular health promotion, and provide empirical support for the localization of chronic disease management within China.

## Data Availability

The original contributions presented in the study are included in the article/supplementary material, further inquiries can be directed to the corresponding authors.

## References

[1] Zhang L Ji H Huang Y Hu H Li B Yang Y . Association of BAX hypermethylation with coronary heart disease is specific to individuals aged over 70. Medicine (Baltimore). (2019) 98:e14130. doi: 10.1097/MD.0000000000014130, 30681575 PMC6358363

[2] Zhao D. Why dentists need to learn the epidemiological status and prevention strategy of coronary heart disease in China. Zhonghua Kou Qiang Yi Xue Za Zhi. (2016) 51:385–6. doi: 10.3760/cma.j.issn.1002-0098.2016.07.001 27480425

[3] Libby P. The changing landscape of atherosclerosis. Nature. (2021) 592:524–33. doi: 10.1038/s41586-021-03392-8, 33883728

[4] Osokpo OH Lewis LM Ikeaba U Chittams J Barg FK Riegel B. Self-Care of African Immigrant Adults with chronic illness. Clin Nurs Res. (2022) 31:413–25. doi: 10.1177/10547738211056168, 34726102 PMC8951348

[5] Riegel B Jaarsma T Lee CS Strömberg A. Integrating symptoms into the middle-range theory of self-Care of Chronic Illness. ANS Adv Nurs Sci. (2019) 42:206–15. doi: 10.1097/ANS.0000000000000237, 30475237 PMC6686959

[6] Jaarsma T Cameron J Riegel B Stromberg A. Factors related to self-Care in Heart Failure Patients According to the middle-range theory of self-Care of Chronic Illness: a literature update. Curr Heart Fail Rep. (2017) 14:71–7. doi: 10.1007/s11897-017-0324-1, 28213768 PMC5357484

[7] Babygeetha A Devineni D. Social support and adherence to self-care behavior among patients with coronary heart disease and heart failure: a systematic review. Eur J Psychol. (2024) 20:63–77. doi: 10.5964/ejop.12131, 38487598 PMC10936663

[8] Durante A Younas A Cuoco A Boyne J Rice BM Juarez-Vela R . Burden among informal caregivers of individuals with heart failure: a mixed methods study. PLoS One. (2023) 18:e0292948. doi: 10.1371/journal.pone.0292948, 37976279 PMC10656022

[9] Vellone E Riegel B Alvaro R. A situation-specific theory of caregiver contributions to heart failure self-care. J Cardiovasc Nurs. (2019) 34:166–73. doi: 10.1097/JCN.0000000000000549 30363017

[10] Caggianelli G Alivernini F Chirico A Iovino P Lucidi F Uchmanowicz I . The relationship between caregiver contribution to self-care and patient quality of life in heart failure: a longitudinal mediation analysis. PLoS One. (2024) 19:e0300101. doi: 10.1371/journal.pone.0300101, 38470867 PMC10931462

[11] Vellone E Riegel B Cocchieri A Barbaranelli C D'Agostino F Glaser D . Validity and reliability of the caregiver contribution to self-care of heart failure index. J Cardiovasc Nurs. (2013) 28:245–55. doi: 10.1097/JCN.0b013e318256385e, 22760172

[12] Buck HG Harkness K Wion R Carroll SL Cosman T Kaasalainen S . Caregivers' contributions to heart failure self-care: a systematic review. Eur J Cardiovasc Nurs. (2015) 14:79–89. doi: 10.1177/1474515113518434, 24399843

[13] Luttik ML Jaarsma T Moser D Sanderman R van Veldhuisen DJ. The importance and impact of social support on outcomes in patients with heart failure: an overview of the literature. J Cardiovasc Nurs. (2005) 20:162–9. doi: 10.1097/00005082-200505000-00007, 15870586

[14] Buck HG Stromberg A Chung ML Donovan KA Harkness K Howard AM . A systematic review of heart failure dyadic self-care interventions focusing on intervention components, contexts, and outcomes. Int J Nurs Stud. (2018) 77:232–42. doi: 10.1016/j.ijnurstu.2017.10.007, 29128777 PMC7059555

[15] Gohari F Hasanvand S Gholami M Heydari H Baharvand P Almasian M. Comparison of the effectiveness of home visits and telephone follow-up on the self-efficacy of patients having un-dergone coronary artery bypass graft surgery (CABG) and the burden of their family caregivers: a randomized con-trolled trial. Invest Educ Enferm. (2022) 40:e14. doi: 10.17533/udea.iee.v40n1e14, 35485627 PMC9052712

[16] Verweij L Jørstad HT Minneboo M ter Riet G Peters RJG Scholte op Reimer WJM . The influence of partners on successful lifestyle modification in patients with coronary artery disease. Int J Cardiol. (2021) 332:195–201. doi: 10.1016/j.ijcard.2021.04.007, 33823215

[17] Wang E Zhang J Peng S Zeng B. The association between family function and adolescents’ depressive symptoms in China: a longitudinal cross-lagged analysis. Front Psych. (2021) 12:744976. doi: 10.3389/fpsyt.2021.744976, 34975563 PMC8718401

[18] Ahn S Romo RD Campbell CL. A systematic review of interventions for family caregivers who care for patients with advanced cancer at home. Patient Educ Couns. (2020) 103:1518–30. doi: 10.1016/j.pec.2020.03.012, 32201172 PMC7311285

[19] Bolgeo T Di Matteo R Simonelli N Dal Molin A Bassola B Lusignani M . Psychometric testing of the caregiver contribution to self-care of coronary heart disease inventory. PLoS One. (2024) 19:e0302891. doi: 10.1371/journal.pone.0302891, 38728276 PMC11086860

[20] Dickson VV Iovino P De Maria M Vellone E Alvaro R Di Matteo R . Psychometric testing of the self-care of coronary heart disease inventory version 3.0. J Cardiovasc Nurs. (2023) 38:E131–40. doi: 10.1097/JCN.0000000000000952, 36288481

[21] De Maria M Iovino P Lorini S Ausili D Matarese M Vellone E. Development and psychometric testing of the caregiver self-efficacy in contributing to patient self-care scale. Value Health. (2021) 24:1407–15. doi: 10.1016/j.jval.2021.05.003, 34593163

[22] Lv Q Zhang X Wang Y Xu X Zang X. Cross-cultural adaptation and validation of the caregiver self-efficacy in contributing to patient self-care scale in China. BMC Public Health. (2024) 24:1977. doi: 10.1186/s12889-024-19534-2, 39049013 PMC11267960

[23] Clark NM Rakowski W. Family caregivers of older adults: improving helping skills. Gerontologist. (1983) 23:637–42. doi: 10.1093/geront/23.6.637, 6662379

[24] Lee RL Mok ES. Evaluation of the psychometric properties of a modified Chinese version of the caregiver task inventory--refinement and psychometric testing of the Chinese caregiver task inventory: a confirmatory factor analysis. J Clin Nurs. (2011) 20:3452–62. doi: 10.1111/j.1365-2702.2011.03729.x, 21707805

[25] Brislin RW. Comparative researchmethodology: cross-cultural studies. Int J Psychol. (1976) 11:215–29. doi: 10.1080/00207597608247359

[26] Self Care Measures. (n.d.) Instrument translation process. Available online at: https://self-care-measures.com/instrument-translation-process/ (Accessed February 1, 2025).

[27] Mokkink LB Terwee CB Patrick DL Alonso J Stratford PW Knol DL . The COSMIN checklist for assessing the methodological quality of studies on measurement properties of health status measurement instruments: an international Delphi study. Qual Life Res. (2010) 19:539–49. doi: 10.1007/s11136-010-9606-8 20169472 PMC2852520

[28] Schofield P Gough K Lotfi-Jam K Aranda S. Validation of the supportive care needs survey-short form 34 with a simplifie desponse format in men with prostate cancer. Psychooncology. (2012) 21:1107–12. doi: 10.1002/pon.2016, 21800397

[29] Streiner DL Norman GR. Health Measurement Scales: A Practical Guide to their Development and Use. Oxford: Oxford University Press (1995).

[30] Zheng C Liu F Zheng Y Chen P Zhou M Zhang H. Psychometric properties of the Chinese version of the self-care scale for older adults undergoing hip fracture surgery: a translation and validation study. Front Public Health. (2023) 11:1119630. doi: 10.3389/fpubh.2023.1119630, 37006555 PMC10050582

[31] Wild D Grove A Martin M Eremenco S McElroy S Verjee-Lorenz A . Principles of good practice for the translation and cultural adaptation process for patient-reported outcomes (PRO) measures: report of the ISPOR task force for translation and cultural adaptation. Value Health. (2005) 8:94–104. doi: 10.1111/j.1524-4733.2005.04054.x, 15804318

[32] Lynn MR. Determination and quantification of content validity. Nurs Res. (1986) 35:382–5. doi: 10.1097/00006199-198611000-00017, 3640358

[33] Davis LL. Instrument review: getting the most from your panel ofexperts. Appl Nurs Res. (1992) 5:194–7. doi: 10.1016/S0897-1897(05)80008-4

[34] Kline RB. Principles and Practice of Structural Equation Modeling. 4th ed. New York, NY: The Guilford Press (2016).

[35] Taherian R Jalali-Farahani S Karimi M Amiri P Maghsoudi E Mirmiran P . Factors associated with pre-hypertension among Tehranian adults: a novel application of structural equation models. Int J Endocrinol Metab. (2018) 16:e59706. doi: 10.5812/ijem.59706, 30197658 PMC6113714

[36] Beaton DE Bombardier C Guillemin F Ferraz MB. Guidelines for the process of cross-cultural adaptation of self-report measures. Spine (Phila Pa 1976). (2000) 25:3186–91. doi: 10.1097/00007632-200012150-00014, 11124735

[37] Vixner L Hambraeus K Äng B Berglund L. High self-reported levels of pain 1 year after a myocardial infarction are related to long-term all-cause mortality: a SWEDEHEART study including 18 376 patients. J Am Heart Assoc. (2023) 12:e029648. doi: 10.1161/JAHA.123.029648, 37584219 PMC10547330

[38] Yang T Zhu Z Barnett R Zhang W Jiang S. Tobacco advertising, anti-tobacco information exposure, environmental smoking restrictions, and unassisted smoking cessation among Chinese male smokers: a population-based study. Am J Mens Health. (2019) 13:1557988319856152. doi: 10.1177/1557988319856152, 31185783 PMC6563409

[39] Riegel B Carlson B Moser DK Sebern M Hicks FD Roland V. Psychometric testing of the self-care of heart failure index. J Card Fail. (2004) 10:350–60. doi: 10.1016/j.cardfail.2003.12.001, 15309704

[40] McCrae RR Kurtz JE Yamagata S Terracciano A. Internal consistency, retest reliability, and their implications for personality scale validity. Personal Soc Psychol Rev. (2011) 15:28–50. doi: 10.1177/1088868310366253, 20435807 PMC2927808

[41] Bahcecioglu Turan G Karaaslan F Özer Z. The effects of nutrition education with Pecha Kucha method on prevention of malnutrition in cancer patients undergoing radiotherapy: a randomised controlled study. BMC Cancer. (2025) 25:1355. doi: 10.1186/s12885-025-14626-7, 40847291 PMC12372213

[42] Lee J Kim K Vellone E Park S. Caregiver self-efficacy in contributing to patient self-care (CSE-CSC) scale: psychometric testing in a population of caregivers of Parkinson’s disease in the Republic of Korea. Appl Nurs Res. (2025) 84:151981. doi: 10.1016/j.apnr.2025.151981, 40592654

[43] Locatelli G Iovino P Jurgens CY Alvaro R Uchmanowicz I Rasero L . The influence of caregiver contribution to self-care on symptom burden in patients with heart failure and the mediating role of patient self-care: a longitudinal mediation analysis. J Cardiovasc Nurs. (2024) 39:255–65. doi: 10.1097/JCN.0000000000001024, 37550831

[44] Kline R Kline RB. Principles and practice of structural equation modeling. J Am Stat Assoc. (2011) 101. doi: 10.1002/0470013192.bsa655

[45] Sousa VD Rojjanasrirat W. Translation, adaptation and validation of instruments or scales for use in cross-cultural health care research: a clear and user-friendly guideline. J Eval Clin Pract. (2011) 17:268–74. doi: 10.1111/j.1365-2753.2010.01434.x, 20874835

